# Challenges in Treating Genodermatoses: New Therapies at the Horizon

**DOI:** 10.3389/fphar.2021.746664

**Published:** 2022-01-05

**Authors:** Marie-Anne Morren, Eric Legius, Fabienne Giuliano, Smail Hadj-Rabia, Daniel Hohl, Christine Bodemer

**Affiliations:** ^1^ Pediatric Dermatology Unit, Departments of Dermatology and Venereology and Pediatrics, University Hospital Lausanne, University of Lausanne, Lausanne, Switzerland; ^2^ Department for Human Genetics, University Hospitals Leuven, KU Leuven, ERN Genturis and ERN Skin, Leuven, Belgium; ^3^ Department of Medical Genetics, University Hospital Lausanne, Lausanne, Switzerland; ^4^ Department of Pediatric Dermatology and Dermatology, National Reference Centre for Genodermatosis and Rare Diseases of the Skin (MAGEC), Hôpital Necker-Enfants Malades, and Assistance Publique-Hôpitaux de Paris, Université Paris Descartes, ERN Skin, Paris, France; ^5^ Department of Dermatology and Venereology, University Hospital Lausanne, University of Lausanne, Lausanne, Switzerland

**Keywords:** genodermatoses, unmet medical needs, reoriented drugs, cell therapy, genetic engeneering, personalised medicine

## Abstract

Genodermatoses are rare inherited skin diseases that frequently affect other organs. They often have marked effects on wellbeing and may cause early death. Progress in molecular genetics and translational research has unravelled many underlying pathological mechanisms, and in several disorders with high unmet need, has opened the way for the introduction of innovative treatments. One approach is to intervene where cell-signaling pathways are dysregulated, in the case of overactive pathways by the use of selective inhibitors, or when the activity of an essential factor is decreased by augmenting a molecular component to correct disequilibrium in the pathway. Where inflammatory reactions have been induced by a genetically altered protein, another possible approach is to suppress the inflammation directly. Depending on the nature of the genodermatosis, the implicated protein or even on the particular mutation, to correct the consequences or the genetic defect, may require a highly personalised stratagem. Repurposed drugs, can be used to bring about a “read through” strategy especially where the genetic defect induces premature termination codons. Sometimes the defective protein can be replaced by a normal functioning one. Cell therapies with allogeneic normal keratinocytes or fibroblasts may restore the integrity of diseased skin and allogeneic bone marrow or mesenchymal cells may additionally rescue other affected organs. Genetic engineering is expanding rapidly. The insertion of a normal functioning gene into cells of the recipient is since long explored. More recently, genome editing, allows reframing, insertion or deletion of exons or disruption of aberrantly functioning genes. There are now several examples where these stratagems are being explored in the (pre)clinical phase of therapeutic trial programmes. Another stratagem, designed to reduce the severity of a given disease involves the use of RNAi to attenuate expression of a harmful protein by decreasing abundance of the cognate transcript. Most of these strategies are short-lasting and will thus require intermittent life-long administration. In contrast, insertion of healthy copies of the relevant gene or editing the disease locus in the genome to correct harmful mutations in stem cells is more likely to induce a permanent cure. Here we discuss the potential advantages and drawbacks of applying these technologies in patients with these genetic conditions. Given the severity of many genodermatoses, prevention of transmission to future generations remains an important goal including offering reproductive choices, such as preimplantation genetic testing, which can allow selection of an unaffected embryo for transfer to the uterus.

## Introduction

Genodermatoses are rare monogenic diseases that affect less than 1/2000 people, which primarily manifest as skin abnormalities, yet they are commonly associated with systemic symptoms (Aşkın et al., 2020). Some conditions may induce neonatal mortality including Harlequin ichthyosis (HI), severe generalised junctional epidermolysis bullosa (EB), severe hypohidrotic ectodermal dysplasia (HED), Netherton syndrome (NS), as well as ankyloblepharon ectodermal defects cleft lip palate (AEC) syndrome. In many life expectancy is reduced or an enhanced cancer risk is present, as observed in xeroderma pigmentosum (XP), neurofibromatosis type1 (NF1), naevoid basal cell nevus syndrome or junctional (JEB), and dystrophic (DEB) EB. Most of these diseases also markedly affect patient quality of life (Aşkın et al., 2020). While treatment modalities are still limited, some of them evolve rapidly in regard to secondary inflammatory or oncologic consequences. At this moment, treatment options are for most of these diseases restricted to skin and wound care, comprising compounded topical preparations, surgery, symptomatic pain relief, treatment of itching, as well as treatment of complications (Bardhan et al., 2020; Mazereeuw-Hautier et al., 2019; Mazereeuw-Hautier et al., 2019; Bodemer et al., 2021; Lenders and Brand, 2021) like skin cancers (de Andrade et al., 2021). This is complemented with patient education designed to enhance disease understanding and therapy adherence, as well as to teach how to avoid triggering factors, including heat and sun exposure among others (de Andrade et al., 2021; Dufresne et al., 2013). Only very few systemic drugs are available and if so, undesirable effects must be taken into account, given that treatment may be required from birth or an early age on, as in the case of retinoid administration. The socio-economic burden of most diseases is very high. In a survey involving six European countries, the average annual costs for EB varied from country to country. These costs ranged from €9,509 to €49,233 in the reference year 2012, 18% of which were direct medical costs, 74,8% non-healthcare costs (non-healthcare transportation, social care services, and caregiver’s time), and 7.2% related to productivity losses (Angelis et al., 2016).

During the 21st century, a revolution took place that consisted in unravelling the molecular genetic background of genodermatoses, especially with the advent of high throughput sequencing techniques. This enabled the thorough investigation of underlying pathophysiological mechanisms, which in turn opened ways to new treatment modalities. This paper provides an overview of these new treatment strategies. In addition, we review the different ongoing efforts that seek to repair the causative genetic defects, yet without paying too much attention to technical features. Besides, we also present some treatment illustrations, some of which have already entered the clinical trial stage.

## Interfering With the Pathophysiological Pathways Disturbed by the Mutated Gene

### Intervening in Disease Pathways

Over-activation of the involved pathway may be caused by a gain of function (GOF) of a main protein or, alternatively, by loss of function (LOF) of an inhibiting protein. To illustrate, Costello syndrome is caused by GOF mutations in the *Harvey rat sarcoma viral oncogene homolog (HRAS)* gene, whereas neurofibromatosis Type 1 is due to LOF mutations in *NF1* gene (Walker and Upadhyaya, 2018). Both genetic variants result in an overactive mitogen-activated protein kinase (MAPK) pathway, commonly called RASopathies. Inhibiting this overactive pathway is thus a logical option, with similar drugs possibly used for different RASopathies. However, the overactive RAS proteins resulting from LOF variants in *NF1* might activate several downstream pathways. This may be tissue-dependent, as hypothesised in NF-1; as a result, this requires targeting different manifestations, separately. Hence, the optimal treatment for neurofibromas may be different from those involving skeletal abnormalities or cognitive dysfunction (Walker and Upadhyaya, 2018). Fortunately, for many pathways, drugs have already been developed in the framework of other pathologies. Commonly, these are often anti-cancer drugs that may be repurposed for use in genodermatoses, provided that undesirable effects are acceptable.

A recent example is the orphan genodermatosis Olmsted syndrome. It is a rare form of painful mutilating palmoplantar keratoderma (PPK), presenting early in life with periorificial keratotic plaques, which are at times associated with alopecia, along with a risk to develop spinocellular carcinomas later in life. The cause in most patients is a heterozygous GOF mutation in *transient receptor potential vanniloid-3 (TRPV3)*, which encodes Ca^2+^ permeable channels. The latter form a signalling complex with transforming growth factor-α (TGF-α) and epidermal growth factor receptor (EGFR). Over-activation of the channels stimulates TGF-α release, which in turn is a stimulating ligand for EGFR. This results in an increased channel activity, hence creating a positive feed-back loop. In a proof of concept study, three patients with activating *TRPV3* mutations were treated using erlotinib, an EGFR-blocker (Greco et al., 2020). Within 3 months after initiating therapy, PPK had drastically improved and pain disappeared.

Another illustration is Gorlin syndrome, also referred to as nevoid basal cell carcinoma (BCC) syndrome, which is caused by LOF variants in the tumour suppressor patched-1 homolog gene (*PTCH1*), provoking release of smoothened (SMO) oncogene inhibition. This results in the subsequent re-activation of the Hedgehog-signalling pathway. Vismodegib, which is a SMO inhibitor, was able to reduce not only the tumour burden and induction of new BCC, but also the growth of jaw keratocystic odontogenic tumours in Gorlin patients (Booms et al., 2015).

Intervening within a pathway that is less active or even inactive appears to be more challenging. DNA-repair disorders are caused by a defective DNA-repair pathway that was initially aimed at restoring genomic damage. As a result, naturally occurring DNA lesions are rapidly neutralized. If this pathway is defective, this predisposes to cancer. Only very few therapeutic possibilities are available for these diseases (de Andrade et al., 2021) (Weon and Glass, 2019). Screening of a library comprising Food and Drug Administration (FDA)-approved drugs has shown that the sulfonylureas acetohexamide and glimepiride are able *in vitro* to enhance viability in XPA cells after UV radiation at rather high dosages (Mazouzi et al., 2017). The exact mechanism is not yet understood. Moreover, preliminary data suggest that nicotinamide or other SIRT-1 inhibitors could be of benefit for XPD/CS patients by decreasing not only UV damage but also preventing neurological degradation. Nevertheless, further studies are needed to confirm these encouraging results (Vélez-Cruz et al., 2013).

Erythropoietic protoporphyria is most often caused by a biallelic loss of function mutation in the gene encoding ferrochelatase, thereby abolishing normal haem production and resulting in the accumulation of protoporphyrin in erythroid cells. The accumulated phototoxic protoporphyrin in superficial vessels is activated by blue light. From early childhood on, severe neuropathic pain, followed by oedema and blistering develops within minutes after sun exposure, which obliges patients to avoid any sun exposure. A recent therapeutic approach is the stimulation of photoprotective eumelanin production by stimulating the melanocortin 1 receptor in melanocytes using its agonist afamelanotide. The latter is a potent analogue of human α-melanocyte-stimulating hormone. Clinically, this approach has been shown to enable symptom-free sun exposure, thereby improving patients quality of life (Langendonk et al., 2015).

### Targeted Therapies for Components of Inflammatory Pathways

The genetic defects underlying genodermatoses likely induce an inflammatory reaction cascade, which contributes to the phenotypic presentation.

The hereditary ichthyoses are a large group of various diseases presenting with hyperkeratosis, scaling, and mostly different degrees of inflammation that involves the entire skin. These conditions are manifest from birth on; in rare cases, they are associated with other organ involvements (Mazereeuw-Hautier et al., 2019). This entity is a diverse group, with more than 50 genes so far identified as the underlying cause. Phenotypically, these conditions present as ichthyosis vulgaris, recessive X-linked, epidermolytic, autosomal recessive congenital ichthyosis (ARCI), and NS, among others. To date, treatment is largely focussed on targeting hyperkeratosis with topical agents and retinoids (Mazereeuw-Hautier et al., 2019). Besides, anti-inflammatory treatments based on corticosteroids and calcineurin inhibitors are commonly employed so as to control inflammation (Mazereeuw-Hautier et al., 2019). More recently, biotherapies targeting the inflammatory process, including TNF-α inhibitors and omalizumab, have been used with some success (Fontao et al., 2011; Yalcin, 2016; Roda et al., 2017).

Surprisingly, a study evaluating the immune dysregulation in patients suffering from ichthyosis identified a large number of ichthyosis subtypes that share IL-23/Th17 skewing in the skin (Paller et al., 2017). This observation offered a more solid pathophysiological basis for new treatment targets. Ustekinumab is a human monoclonal antibody that binds to the p40 subunit common to both Il-12 and Il-23. This agent thus blocks the binding of these cytokines to their receptor, and it has been successfully applied in a patient suffering from erythrodermic ichthyosis caused by a biallelic pathogenic variant in *NIPAL4* (Poulton et al., 2019). This agent has also been employed with success in a patient with NS (Volc et al., 2020), and in another patient exhibiting a biallelic mutation in *desmoplakin* that manifested as ichthyosis (Paller et al., 2018). Different case reports and small series have already been published focussing on Il-17 inhibitors such as sekukinumab (Blanchard and Prose, 2020; Luchsinger et al., 2020; Barbieux et al., 2021) in NS, although sustained improvement could not be obtained in one patient. Given that, after this treatment, a Th2 signature remained (Barbieux et al., 2021), using dupilumab, a blocker of the IL-4 receptor’s alpha-chain, thereby blocking Il-4 and Il-13 cytokines, could be another target for some NS patients, as it has already been tested with encouraging results (Andreasen et al., 2020; Steuer and Cohen, 2020; Süßmuth et al., 2021).

A recent paper identified potential therapeutic targets for Harlequin ichthyosis (HI), which is the most severe form of ichthyosis, and it is often associated with marked neonatal lethality. In an *in vitro* model, investigators discovered an upregulation of IL-36α and IL-36γ, as well as of STAT1 and its downstream target, consisting of inducible nitric oxide synthase (NOS2). Treatment using either a NOS2-inhibitor or the JAK inhibitor tofacitinib was proven able to restore the lipid barrier in the HI 3D-model (Enjalbert et al., 2020). Drugs blocking either Il-36 or downstream IL-23 and IL-17 would probably be an option that deserves to be considered. Il-36 cytokines are secreted by keratinocytes, representing a danger signal. Hence, we believe this cytokine to be a potential candidate, given that it is one of the earliest cytokines that are being secreted in response to barrier disruption, as seen in ichthyosis.

However, more large-scale studies are now needed, seeking to evaluate the effect of these different biologicals and identify the precise patient profile that would best respond to the different drugs. So far, several trials have already been initiated (see Table 1).

**TABLE 1:** New and repurposed drugs for different genodermatoses, based on pathophysiology (ongoing trials mentioned in clinicaltrials.gov with NCT).

Disease	Gene	Function/pathway	Secondary pathway’s	Drugs under investigation
Congential Hemidysplasia with Ichthyosiform erythroderma and Limb Defects (CHILD syndrome) Paller et al. (2011)	*NAD(P)H steroid dehydrogenase-like (NSDHL)*	Cholesterol biosynthetic pathway		Lovastatin/cholesterol cream
Epidermolysis bullosa general Kern et al. (2019)				Betulin (Oleogel-10°)
				NCT03068780
				(SD-101-0.0) allantoin cream (Alwextin) NCT02384460
				Diacerein
				NCT 03472287
				AC-203
				NCT 03468322
EB simplex	*KRT14/KRT5*	Collapse of keratin network in basal layer		Topical sirolimus 2%
				NCT02960997
				Awaiting results
EB simplex Kerns et al. (2007)	*KRT14, (KRT5)*	Collapse of keratin network in basal layer	Nrf2 signalling	Sulforaphane + diarylpropionitrile
				Broccoli sprout NCT02592954
EB simplex Swartling et al. (2010)	*KRT14/KRT5*	Collapse of keratin network in basal layer		Botulinum toxin
				NCT03453632
EB simplex Castela et al. (2019)	*KRT14/KRT5*	Collapse of keratin network in basal layer	Chelliah et al. (2018) Th-1 and Th-17 activation	apremilast
EB simplex, Severe generalized Limmer et al. (2019); Wally et al. (2018)	*KRT14*	Collapse of keratin network in basal layer	Il-1b signaling	Diacerein
				NCT03389308
EB pruriginosa dystrophic Shehadeh et al. (2020); Zhou et al. (2021)	*COL7A1*	Defective anchoring fibrils at the DEJ		Dupilumab
EB dystrophic Schräder et al. (2019)	*COL7A1*			Cannabidiol CBD
EB dystrophic Guttmann-Gruber et al. (2018)	*COL7A1*			Topical calcipotriol 0,5 mg/g
EB generalized dystrophic Nyström et al. (2015)	*COL7A1*	Defective anchoring of BM to dermis	TGF-β signaling	Losartan
Erythrokeratodermia-cardiomyopathy (desmosomal disorder) Paller et al. (2018)	*DSP*	Desmosomal detachement	Overactive Th17- Th22 and Th1 axis	sekukinumab
Gorlin syndrome Booms et al. (2015); Tang et al. (2012); Goldberg et al. (2011)	*PTCH1*	Hedgehog pathway		Hedgehog inhibitors like vismodegib
Ichthyosis	*Different genes*	Epidermal barrier dysfunction	Overactive Th17- Th22 axis	sekukinumab (anti IL-17) is awaiting results NCT03041038 Paller (2019)
Ichthyosis	*Different genes*	Epidermal barrier dysfunction	Overactive Th17- Th22 axis and Il-36	imsidolimab (ANB019, anti IL-36R) NCT04697056
Autosomal recessive congenital ichthyosis (ARCI)	*Different genes*	Epidermal barrier dysfunction	Overactive Th17- Th22 axis	a study is yet to start with ustekinumab (anti IL-12/IL-23) NCT04549792
	*NIPAL4* Poulton et al. (2019)			
Ichthyosis (Harlequin) Enjalbert et al. (2020)	*ABCA12*	Epidermal barrier dysfunction	Il-36 overexpression	Tofacitinib
Ichthyosis lamellar	*TGM-1*	Epidermal barrier dysfunction		epigallocatechin-3-gallate (veregen)
				NCT01222000
Netherton disease Liddle et al. (2021)	*Spink5*	multidomain serine protease inhibitor expressed in stratified epithelial tissue, inhibits cleavage of a.o. desmosomes		Kallikrein-5 inhibitor
				But : TGM1 like domains perhaps not targeted Wiegmann et al. (2019)
Netherton disease Fontao et al. (2011); Roda et al. (2017)	*Spink5*	multidomain serine protease inhibitor expressed in stratified epithelial tissue, inhibits cleavage of a.o. desmosomes	Overactive Th17- Th22 axis and high TNFα expression	TNFα-blockers Infliximab
				Adalimumab NCT02113904
Netherton disease Yalcin (2016)	*Spink5*	multidomain serine protease inhibitor expressed in stratified epithelial tissue, inhibits cleavage of a.o. desmosomes	High total and specific IgE	Omalizumab
Netherton disease Blanchard and Prose (2020); Luchsinger et al. (2020); Barbieux et al. (2021)	*Spink5*	multidomain serine protease inhibitor expressed in stratified epithelial tissue, inhibits cleavage of a.o. desmosomes	Overactive Th17- Th22 axis, high total and specific IgE	Il-17 blockers
				Sekukinumab, Ixekizumab
Netherton disease Volc et al. (2020)	*Spink5*	multidomain serine protease inhibitor expressed in stratified epithelial tissue, inhibits cleavage of a.o. desmosomes	Overactive Th17- Th22 axis, high total and specific IgE	Il-12/Il-23 blockers Ustekinumab
Netherton disease Andreasen et al. (2020); Steuer and Cohen (2020); Süßmuth et al. (2021)	*Spink5*	multidomain serine protease inhibitor expressed in stratified epithelial tissue, inhibits cleavage of a.o. desmosomes	Persistence of Th2 signature after treatment with ixekizumab	Dupilumab (anti IL-4/IL13)
				NCT04244006
Neurofibromatosis 1 Walker and Upadhyaya (2018)	*Neurofibromin*	RAS/MAPK	PI3K/AKT/mTOR	Inhibitors of MEK, B-RAF, m-TOR, TGF-β, RTK’s (VEGFR, KIT, MET, PDGFR), JAK-STAT, RAS
Neutral lipid storage disease	LOF mutations in *ABHD5 or PNPLA2 *	defective catabolic pathway of triacylglycerols resulting in systemic accumulation of triglycerides	PPAR activation	Fibrates
				NCT01527318
Olmsted syndrome Greco et al. (2020)	*TRPV3*	TRPV3/TGFa/EGFR		Erlotinib (EGFR-inh)
P63-related ectodermal dysplasia Aberdam et al. (2020)	*P63*	Master regulator of embryonic steps of epithelial development		PRIMA-1MET,
Pachyonychia congenita Zhao et al. (2011); Abdollahimajd et al. (2019); Frommherz and Has (2020)	*KRT6A,*	Collapse of keratin network in palmoplantar skin		Statins (*KRT6A inhibit promotor activity)*
Pachyonychia congenita Kerns et al. (2018)	*KRT16, 17*	Collapse of keratin network in palmoplantar skin	Nrf2 signalling	Sulforaphane + diarylpropionitrile
				Broccoli sprout NCT02592954
Pachyonychia congenita Teng et al. (2018); Daroach et al. (2019)	*KRT6A,6B,6C,16,17*	Collapse of keratin network in palmoplantar skin		Sirolimus (topical or oral)
				QTORIN™ 3.9% rapamycin (sirolimus) anhydrous gel
				NCT03920228 and NCT03920228
Pachyonychia congenita González-Ramos et al. (2016); Thomas and Sahota (2020); Koren et al. (2020); Lwin et al. (2018)		Collapse of keratin network in palmoplantar skin		Botulinum toxin
Pityriasis Rubra Pilaris Eytan et al. (2014); Lwin et al. (2018)	*CARD14*	activation of NF-κB signaling	interleukin IL-12 and IL-23 are upstream activators of NF-κB signaling	Ustekinumab
Pityriasis Rubra Pilaris Vasher et al. (2010)				TNF-α antagonists
Pityriasis Rubra Pilaris De Felice et al. (2020)				Th-17 antagonists
Porokeratosis Atzmony et al. (2020)		Mevalonate kinase		Lovastatin/cholesterol
Porphyria (erythropoietic protoporphyria)	*FECH*	Haemsynthesis		Afamelanotide Langendonk et al. (2015) NCT04053270
	*ALAS2*			Dersimelagon
				NCT04402489
				NCT05005975
Psoriasis familial early onset Signa et al. (2019)	*CARD 14*	activation of NF-κB signaling	interleukin IL-12 and IL-23 are upstream activators of NF-κB signaling	ustekinumab
Psoriasis pustular Bachelez et al. (2019)	*IL36RA*			Spesolimab NCT04549792, NCT03886246, NCT04399834
PTEN hamartoma tumor syndrome Van Damme et al. (2020); Yehia et al. (2019); Komiya et al. (2019)	*PTEN*	PTEN inhibits		Sirolimus
		PI3K/AKT/mTOR signaling		
PTEN hamartoma tumor syndrome	*PTEN*			Everolimus
PTEN hamartoma tumor syndrome Yehia et al. (2019)	*PTEN*			Miransertib (Synonyms: ARQ-092)
				Akt-inhibitor
Rendu-Osler-Weber disease Van Damme et al. (2020)	*ACVRL1(ALK1)*	transforming growth factor-β superfamily	VEGF signaling	Bevacizumab (VEGF inh.)
	*ENG*	signaling pathway		
	*SMAD4, GDF2*			
Sjogren Larsson Syndrome	LOF fatty aldehyde dehydrogenase *ALDH3A2* key component of the detoxification pathway of aldehydes arising from lipid peroxidation events			Reproxalap cream, which binds and traps free aldehydes
				NCT03445650
				NS2 cream
				NCT02402309
Tuberous sclerosis complex Habib et al. (2016)	*TSC1*	m-TOR		m-TOR inhibitor (Sirolimus, Everolimus)
	*TSC2*			
Xeroderma pigmentosum group A Mazouzi et al. (2017)	*XPA*	nucleotide excision repair (NER) pathway		Acetohexamide
Xeroderma pigmentosum group D/Cockayne Syndrome Vélez-Cruz et al. (2013)	*XPD*	nucleotide excision repair (NER) pathway		Nicotinamide, SIRT-1 inhibitor
**Mosaic disorders**				
AVM Lekwuttikarn et al. (2019)	*KRAS*, *NRAS*, *BRAF*, and *MAP2K1*	RAS-MAPK pathway		MEK inhibitor (Trametinib)
Congenital hemangioma (rapidly involuting, non involuting)	*GNAQ*, *GNA11*	RAS/MAPK		m-TOR inhibitor ?
Lymphatic malformation including generalized lymphatic malformation Hammill et al. (2011) for a review see Geeurickx and Labarque (2021)	*PIK3CA*	PI3K/AKT/mTOR pathway		m-TOR inhibitor (Sirolimus, Everolimus systemic or topical if microcystic only in skin)
Kaposiform hemangioendothelioma, tufted angioma, and Kasabach Merrit Syndrome Hammill et al. (2011); Cheraghlou et al. (2019)	*GNAQ*, *GNA11*	RAS/MAPK (receptor for		m-TOR inhibitor (Sirolimus Everolimus)
Maffucci syndrome Serena Tommasini-Ghelfi (1975)	*IDH1*	Tricarboxic acid cycle		Ivosidenib (IDH1-inhibitor)
	*IDH2*	Epigenic control of gene expression		Enasidenib (IDH2-inibitor)
PROS (PIK3CA-related overgrowth syndromes) Parker et al. (2019)	*IK3CA*	PI3K/AKT/mTOR pathway		m-TOR inhibitor (Sirolimus, Everolimus)
PROS (PIK3CA-related overgrowth syndromes) Venot et al. (2018)	*PIK3CA*	PI3K/AKT/mTOR pathway		Pik3CA inhibitor (Alpelisib)
				Taselisib (TOTEM study in press)
Proteus syndrome Keppler-Noreuil et al. (2019); Biesecker et al. (2020)	*AKT*	PI3K/AKT/mTOR pathway		AKT inhibitor (Miransertib)
Slow flow vascular malformations (including blue rubber bleb nevus syndrome) Hammer et al. (2018); Harbers et al. (2021)	*TIE/TEK*	PI3K/AKT/mTOR pathway (TIE/TEK are tyrosine kinase receptors which stimulate the pathway) mammalian (mechanistic target of rapamycin)		m-TOR inhibitor (Sirolimus Everolimus)
	*PIK3CA*			
	*KRAS, NRAS* Al-Olabi et al. (2018)			

Pustular psoriasis, due to a LOF mutation of *Il36RN* that encodes a receptor antagonist, is another disease candidate for IL-36 antagonists (Gooderham et al., 2019). Interestingly, improvement after one single injection was already observed in a proof-of-concept study involving spesolimab. This compound, which is an Il-36R blocker is investigated in a recent study (NCT02978690), however was, associated with mild to moderate undesirable effects, including infections, fever, or arthralgia, in all the seven patients that were tested. It will certainly be interesting to further assess this compound’s clinical effects and undesirable effects, as well (Bachelez et al., 2019), in larger-scale clinical trials (Choon et al., 2021), (Table1).

Familial pityriasis rubra pilaris (PRP) is commonly caused by heterozygous GOF mutations of *CARD14* (Fuchs-Telem et al., 2012), which is a regulator of nuclear factor-κB (NF-κB-) signalling. As this pathway positively regulates Th17 differentiation, and in turn is regulated by TNF-α, indeed treatment with TNF-α inhibitors (Vasher et al., 2010), Th-17 inhibitors (De Felice et al., 2020) and IL12/IL23 inhibitors have been effective treatments (Eytan et al., 2014; Lwin et al., 2018). As the same *CARD14* mutations have been associated with familial early-onset psoriasis (Craiglow et al., 2018), ustekinumab, namely an Il12/Il23-antagonist, may prove to be effective in these psoriasis patients (Signa et al., 2019).

However, other genodermatoses could also be candidates for targeting inflammatory mediators, provided that inflammation is actually part of their clinical manifestations. To illustrate, severe generalised EB simplex is a genodermatosis that manifests as skin blistering upon minimal trauma. This condition is caused by heterozygous *KRT14* mutations, the pro-inflammatory cytokine Il-1 being strongly expressed. Diacerein is a down-regulator of the Il-1 signalling cascade, which is administered in a cream formulation. This agent has proven to be effective in reducing the number of blisters, without significant undesirable effects (Wally et al., 2018; Limmer et al., 2019). Diacerein is also under investigation for EB which is caused by mutations in other genes. AC-203 is another drug with similar properties, which is currently under investigation (Table 1). Targeting Th-17 cells, as well as TGF-β or Th-2 cytokines represent other therapeutic options (Nyström et al., 2015; Castela et al., 2019; Shehadeh et al., 2020; Zhou et al., 2021).

Table 1 provides a summary of genodermatoses, while also listing the new or repurposed drugs currently under investigation.

## Restoring the Underlying Gene Defect/Product

### Read Through Drugs

**FIGURE 1:**
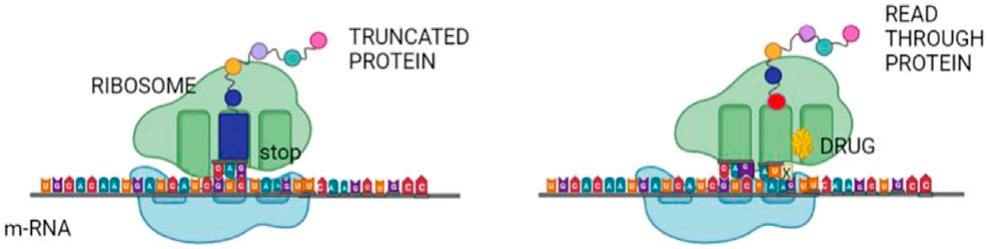
Read through therapy. Nonsense mutations in various genes may be repaired by read-through therapy. Small molecules, known as TR-inducing drugs (TRIDs), enable the translation machinery to suppress a nonsense codon, which would stop further translation of the protein. It allows for the synthesis of a full-length protein with no or only minor disruption of the synthesized protein.

Nonsense mutations leading to premature termination codons (PTCs) and truncated inactive or decoyed proteins are estimated to be the cause of about 11% of all monogenic diseases. Repurposed drugs like aminoglycosides favour, during translation, mispairing of near-cognate tRNA at the place of the premature termination, resulting in the incorporation of a different amino-acid into the translated protein. Usually, this transforms a nonsense mutation into a missense mutation, thereby restoring the full-length protein (Figure 1) (Nagel-Wolfrum et al., 2016).

In genodermatoses, topical gentamycin administration on skin lesions in Hailey-Hailey disease (HHD) (Kellermayer et al., 2006), hereditary hypotrichosis simplex (Peled et al., 2020), and PPK caused by *SERPINB7* mutations (Ohguchi et al., 2018), as well as topical or intradermal (ID) gentamycin administration in dystrophic EB (Woodley et al., 2017) have already been tested, yet with variable results. Moreover, *in vitro* tests in JEB cells were demonstrated to restore expression of laminin 332, (Lincoln et al., 2018), and of XPC in XP cells (Kuschal et al., 2015).

More studies are presently ongoing including topical application GENTELBULL (NCT04644627), with topical BPM 31510 cream (NCT02793960) in EB; intravenous (IV) administration in both JEB (NCT03526159) and DEB (NCT03392909). Trial NCT03012191 in DEB has been completed, with preliminary results showing some C7 expression in exclusively the skin in IV- or ID-treated patients, yet not in topically-treated patients.

Although these readthrough drugs look promising and could be applied in numerous genetic diseases, currently available compounds exhibit weak (less than 5%) activity *in vitro*, and in only a fraction of tested patients. Moreover, the oto- and nephrotoxicity of aminoglycosides is a concern in the event of long-term administration, as is the risk of antibiotic resistance if these drugs are topically applied on chronic wounds, such as in EB. There is an ongoing search for new compounds with a good safety profile or for drug combinations [for a review see (Baradaran-Heravi et al., 2016; Nagel-Wolfrum et al., 2016)]. A recent study investigated amlexanox, which has proven to be effective in cultured fibroblasts and keratinocytes from dystrophic EB patients (Atanasova et al., 2017).

### Protein Therapy

**FIGURE 2:**
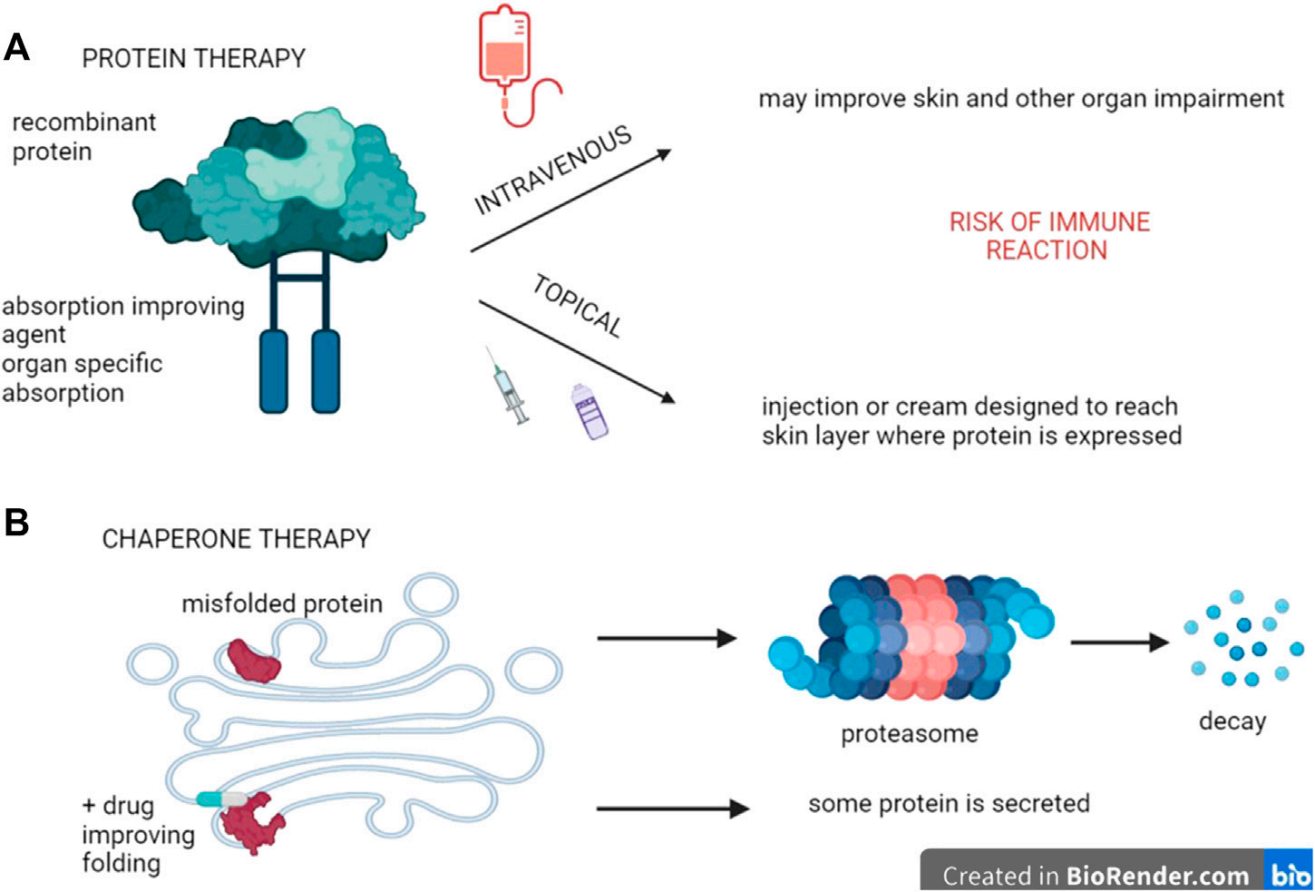
Protein therapy. **(A)** Recombinant proteins can be used to replace dysfunctional ones, provided they are delivered at the site where they are expressed. In genodermatoses, topical application constitutes a possibility that may consist of creams with penetration-improved formulations, intradermal injections, or even intravenous applications if the latter enable the active principle to reach their target environment. **(B)** In some cases, mutations code for proteins that are misfolded, thereby remaining in the endoplasmatic reticulum. Drugs called chaperones that improve this folding are thus likely to circumvent this problem and restore protein activity to some extent.

Replacement of the protein encoded by the mutated gene is currently under investigation in different genodermatoses (Figure 2A). A proof of concept study using a mice model for dystrophic EB, based on missing Type VII collagen (C7) anchoring fibrils, has demonstrated that injecting normal C7 is indeed able to form anchoring fibrils at the dermo-epidermal junction (DEJ). This ability was demonstrated not only in the skin but also in the oesophagus, lasting for several weeks and preventing the mice from dying (Woodley et al., 2013; Hou et al., 2015).

Presently, phase 1 and 2 studies in humans are ongoing, administering IV recombinant C7 in recessive DEB (PTR-01) (NCT03752905 and NCT04599881). Immunogenicity does not appear to be a big issue. However, their size and tendency to form aggregates likely limit skin and other tissue homing. The drug has orphan designation.

Similar ongoing studies are focused on transglutaminase-1 (*TGM-1*)-deficient ARCI. In a skin humanized mouse model, topical application of liposomes containing recombinant human TG1 (rh-TG1) resulted in considerable improvement in ichthyosis phenotype, as well as normalization of the regenerated ARCI skin (Aufenvenne et al., 2013). More recently, such positive outcome was confirmed in a skin equivalent model using thermo-responsive nanogels (tNG) encapsulating TG1 (Witting et al., 2015; Plank et al., 2019).

The timing of protein replacement appears essential, as illustrated in hypohidrotic ectodermal dysplasia (HED), which is a disease that affects several ectodermal structures. Sweat glands, teeth development, and meibomian glands are severely impaired in this condition, with distinctive facial features usually present, as well as sparse blond hair. The condition’s most common cause is a recessive X-linked mutation in *ectodysplasin A* (*EDA*) (Schneider et al., 2018). The inability to sweat may be life-threatening due to hyperthermia.

Fc-EDA, which is a fusion protein linking the constant domain of IgG1 and the receptor-binding portion of EDA, was administered prenatally via amniocentesis into the amniotic cavities of three foetuses of two different pregnancies, namely at Week 26 for one and for the twins at Week 26 and Week 31. Sweat glands, meibomian glands, and tooth germs almost normally developed in the new-borns, being more numerous than in their affected older brothers (Schneider et al., 2018). However, when this Fc-EDA was administered to a baby at birth, no effect was observed, which further underlines that administration must be performed when the pathway is active, meaning between Weeks 20–30 during foetal development for sweat glands. Nevertheless, a larger study with EDI200 administered immediately after birth has been completed, while still awaiting results (NCT01775462).

For genetic diseases caused by retention of the defective protein within the endoplasmatic reticulum, due to misfolding and subsequent absence of transport to the Golgi apparatus, chaperone therapy is under investigation, mostly based on low concentrations of competitive inhibitors. By binding to misfolded enzymes and forming stable complexes instead, the transport and further processing into the Golgi apparatus is being restored. As a result, some (variable) enzymatic activity and partial phenotype rescue is rendered possible (Figure 2B). This technique is under investigation for storage diseases like Gaucher and Fabry disease and also in oculo-cutaneous albinism Type A (OCA1A), (Table 2) (Lenders and Brand, 2021; Teramae et al., 2019), while it may also be taken into consideration for keratinopathies (Chamcheu et al., 2011).

**TABLE 2:** Treatments under investigation aiming to restore protein expression (ongoing trials mentioned in clinicaltrials.gov with NCT)

Disease	Gene	Pathway	Drug	Mechanism
Albinism oculocutaneous type 1B Onojafe et al. (2018); Liu et al. (2021)	*Tyr* (some residual activity)	Melanin metabolism	Nitisinone (mouse model)	Higher levels of tyrosine stabilize tyrosinase
Albinism oculocutaneous type 1A (OCA1A) Teramae et al. (2019) and possibly OCA3 and OCA8	*Tyr type 1* (certain variants causing endoplasmatic reticulum retention) (possibly *Tyrp1* and *Tyrp2*)	Melanin metabolism (retention in ER)	Chaperone therapy low-dose tyrosinase inhibitor like deoxyarbutin (captopril, miconazole)	Target misfolding of tyrosinase, allowing transport from ER/Golgi to melanosome
Dystrophic EB	*COL7A1*		C7	
			NCT03752905 and NCT04599881	
Ectodermal dysplasia anhidrotic Schneider et al. (2018)	*EDA*		Fc-EDA	
Fabry disease Lenders et al. (2021)	*a-galactosidase A*	intracellular accumulation of glycosphingolipids (mainly globotriaosylceramide [Gb3])	*Enzyme replacement therapy (ERT)*	
			IV agalsidase-alfa (Replagal Takeda) or agalsidase-beta (Fabryzyme Genzyme-Sanofi)	
			New generation :	
			Pegunigalsidase NCT03180840; NCT03018730 (longer half life, lower immunogenicity)	
Fabry disease Lenders et al. (2021)	*a-galactosidase A* (check if mutation is suitable at www. galaf oldam enabi lityt able. Com)		*Chaperone therapy* migalastat (improves misfolding)	Target misfolding and transport from ER
Fabry disease Guérard et al. (2017)	*a-galactosidase A*		*substrate reduction therapy reducing accumulation of Gb3 combined with ERT*	Reduction of glycosphingolipid accumulation by inhibiting upstream -located glucosylceramide synthase
			Lucerastat (+ERT)	
			NCT02930655; NCT03737214	
			Venglustat (NCT02228460)	
Lamellar ichthyosis Aufenvenne et al. (2013)	*TGM1*		TG1	
Netherton disease Liddle et al. (2021)	*Spink5*	large multidomain serine protease inhibitor expressed in stratified epithelial tissue	Kallikrein-5 inhibitor Wiegmann et al. (2019)	May replace inhibiting activity but effect on TGM1 like domains to be determined Wiegmann et al. (2019)
Peeling skin disease type 1 Valentin et al. (2020)	Corneodesmosin (*CDSN*)		*CDSN* in liposome-based carrier	
Xeroderma pigmentosum Yarosh et al. (2001)			bacterial DNA repair enzyme, T4 endonuclease V in a liposome formulation	
			NCT00002811	

**TABLE 3:** Cell and gene therapies under investigation for genodermatoses mentioned in clinicaltrials.gov with NCT.

Disease	Study ID number	Type of therapy
*Epidermolysis bullosa*		
		**Cell therapy**
JEB	NCT03490331 hologene 17	*Ex vivo* gene therapy with corrected epidermal stem cells
DEB	NCT04173650	application on wounds of extracellular vesicle (EV) product containing mediators derived from normal allogeneic donor MSCs
	AGLE-102 study	
	UMIN 000028366	Topical applications of human MSCs derived from adipocytes (ALLO-ASC sheet)
		**Gene transfer**
	NCT04186650	*ex vivo gene transfer* using corrected keratinocytes and fibroblasts in skin equivalents
	NCT04227106, NCT02984085 and NCT01263379	*Ex vivo* gene therapy with corrected keratin sheets
	NCT02810951, NCT02493816 and NCT04213261	*Ex vivo* gene therapy with corrected fibroblasts (FC07)
	NCT04186650	*ex vivo* gene therapy with corrected keratinocytes and fibroblasts in skin equivalents
	NCT03536143 and NCT03605069	*in situ* (skin application) gene correction with Beremagene geperpavec gel (KB103)
		**Antisense oligonucleotides**
	NCT03605069	AON targeting exon 73 of COL7A1 RNA in a carbomer-based hydrogel (Q313)
		**Cell therapy**
RDEB (*COL7A1*)	NCT 02579369	Topical Allogeneic MSCs cells on a polyurethane sheet
	ALLU-ASC-DFU	
	NCT04520022	IV Allogeneic Umbilical Cord Blood-derived MSCs
	NCT04153630	IV MSCs derived from bone marrow (BM-MSCs) from a haplo-identical donor
	NCT03529877	IV allogeneic ABCB5+ SCs
	NCT02323789	IV allogeneic MSC
	https://fundingawards.nihr.ac.uk/award/NIHR127963	IV mesenchymal stromal cell infusions
*Fabry disease*		**Gene transfer**
	NCT02800070; NCT03454893	*Ex vivo* gene transfer using a lentivirus carrying *GLA* corrected stem cells
	NCT04046224 and NCT04040049	*in vivo* gene transfer to hepatocytes with *GLA* carrying adeno associated virus
	NCT04519749	*In vivo* gene transfer using a *GLA* carrying adeno-associated virus composed of a capsid with high cardiocyte transducing capacity
*Ichthyosis*		
		**Gene transfer**
ARCI TGM1 deficient	NCT04047732	*in situ* gene transfer with KB105, a replication-incompetent, non-integrating HSV-1 vector expressing human TGM1 formulated as a topical gel
		**Gene transfer**
Netherton Syndrome	NCT01545323	*Ex vivo* gene transfer with autologous epidermal sheets generated from genetically modified skin stem cells
		**Silencing RNA**
*Pachyonychia congenita*	NCT00716014	TD101 targeting N171K in K6A
		**Si-RNA**
*Variegate parophyria*	NCT03338816	A Study to Evaluate the Efficacy and Safety of Givosiran (ALN-AS1) in Patients With Acute Hepatic Porphyrias (AHP), including variegate porphyria

Both enzyme replacement and chaperone therapy are similarly being investigated for the treatment of porphyrias. Nevertheless, the major drawback of these approaches consists in their delivery to the targeted organ (Bustad et al., 2021).

### Cell Therapy

**FIGURE 3:**
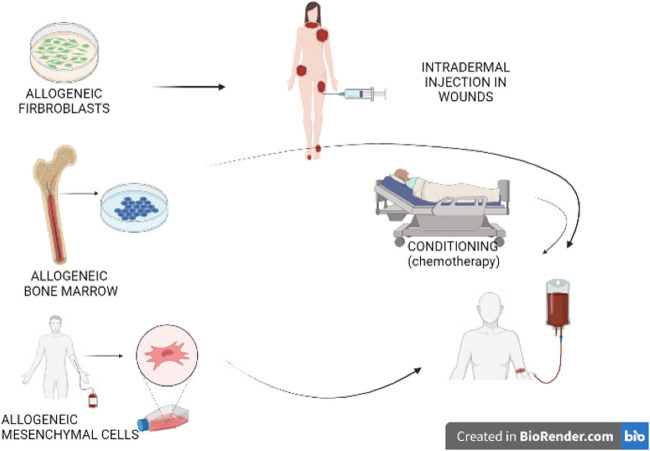
Cell therapy. Allogeneic cells secreting the proteins that are dysfunctional within the patient may improve disease severity. Fibroblasts injected in chronic wounds in dystrophic epidermolysis bullosa are currently under investigation. Bone marrow transplantations improve wound healing and systemic symptoms; nevertheless, this technique requires pre-transplantation conditioning, which is associated with a risk of undesirable effects and even mortality. More recent is the use of allogeneic mesenchymal stem cells, which does not require conditioning. Immune reactions are less common when fibroblasts or mesenchymal stem cells are employed, in comparison with hematopoietic stem cells. These reactions are more common in the event of mutations without spontaneous protein expression in the patient.

Restoring the defective protein by administrating allogeneic cells is another approach in managing genodermatoses (Figure 3). Collagen 7 (C7) is produced by keratinocytes and fibroblasts. Early experiments showed that IV injection of human fibroblasts in athymic mice resulted in the deposition of human C7 that formed anchoring fibrils at the DEJ, yet which occurred in wounded but not in normal mice skin (Woodley et al., 2007). Subsequently, three studies conducted in humans demonstrated ID injection of allogeneic fibroblasts to fasten the healing of wounds in most tested DEB patients (Petrof et al., 2013; Venugopal et al., 2013; Moravvej et al., 2018). Nevertheless, no difference in healing time was observed in comparison with the lesions injected with the vehicle containing albumin and growth factors (Venugopal et al., 2013). Interestingly, the patients who failed in these three studies were unable to express own C7, while the anchoring fibrils seen in the biopsies were rudimentary (Wong et al., 2008). Despite the allogeneic fibroblasts disappearing after 2 weeks, the clinical effect of one single injection lasted 1 month in one study (Petrof et al., 2013), and up to 3 months in another (Moravvej et al., 2018), with C7 expression sustained over 9 months. In contrast with the animal studies, this clearly suggests that the major effect probably consisted of an increase in recipients *COL7A1* mRNA and mutant C7 levels, which was induced by paracrine mediators secreted by the allogeneic fibroblasts (Wong et al., 2008), such as heparin-binding epidermal growth factor-like growth factor (HB-EGF) (Nagy et al., 2011).

Allogeneic bone marrow (BM) transplantation is a technique that was primarily designed to treat hematopoietic malignancies. Later on, this technique was found to be effective in reversing the phenotype of some genetic diseases, primarily comprising primary immune-deficiencies. However, this same approach has also been shown to attenuate the muco-cutaneous manifestations and improve the quality of life of severe JEB and DEB (Wagner et al., 2010). By reducing the intensity of the conditioning regimen, essential undesirable effects and mortality could be reduced (Tolar and Wagner, 2013). Further research allowed for unravelling the potential underlying mechanism (Tamai and Uitto, 2016), thereby refining the technique. Release of high mobility group box 1 protein (HMGB1) from necrotic epithelial cells does transfer signals to bone marrow-derived PDGFRa^+^/CXCR4^+^ cells. This leads to their migration into circulation, along with subsequent homing to the damaged skin through stromal cell-derived factor 1a (SDF-1a)/CXCR4 axis (Tamai and Uitto, 2016). The most essential action of MSCs is likely the secretion of anti-inflammatory agents, whereas they may also differentiate into fibroblasts and keratinocytes. Transplanted patients are able to receive skin grafts from the same donor, which survive for long periods and outgrow into adjacent wounds, thereby suggesting immune tolerance and outgrowth of stem cells (Ebens et al., 2020).

Three open clinical trials have been conducted to date, involving subjects with RDEB using MSCs from healthy donors. Of these, two were carried out in children (Petrof et al., 2015; El-Darouti et al., 2016) and one in adults (Rashidghamat et al., 2020). In these trials two or three IV injections were administered over a 1-month period, without any HLA matching or preconditioning. Effects on wound healing, pain, and especially pruritus seemed to be rather promising, with only mild undesirable effects observed. The latter were mostly transient, and all were unrelated to MSCs. A major concern, however, has been the development of squamous cell carcinomas (SCC) in two out of 10 adult participants during the study period (Rashidghamat et al., 2020). Nevertheless, the causality of this serious event is still being debated, given that SCCs represent a naturally occurring complication of RDEB. No increase in C7 or anchoring fibrils at the DEJ could be detected in skin biopsies. The improvement in adhesion has been attributed to either an increased expression of other junctional adhesion proteins or a reduction in inflammatory mediators that impair DEJ adhesion. Optimizing the number and frequency of MSC infusions, while addressing specific MSC subpopulations like skin homing ATP-binding or cassette subfamily B member 5 positive (ABCB5) MSC (Riedl et al., 2021) are currently being studied and designed to improve the outcome results. Injection of MSC-derived anti-inflammatory products carried in exosomes (Perdoni et al., 2014; Shabbir et al., 2015; McBride et al., 2018) is similarly under investigation, as are HMGB1 and SDF1 able to attract MSC.

### Gene Therapy

In principle, all genodermatoses are candidates for gene therapy (Ain et al., 2021).

The major obstacle to this process is the efficient, safe, and targeted delivery of the gene (Ain et al., 2021; Bustad et al., 2021). Non-integrating viral vectors like adenoviruses were the first to be applied and have so far been the most widely used. Nevertheless, they carry a restricted cargo potential, which may be a limiting factor for large genes such as *NF1* (Walker and Upadhyaya, 2018). Besides, herpesviruses are commonly chosen for *in vivo* transfection. Retroviruses and the subgenus lentiviruses, as well, have the capacity to integrate into the genome, thereby allowing for stable and long-term gene expression, yet with a risk of being mutagenic at the insertion site.

Alternatives to viruses are lipid based nanoparticles (LNPs) and polymeric nanoparticles. These have the advantage of being able to overcome proteolytic degradation in the skin, while they are devoid of mutagenic risk and permit the transport of larger fragments. Moreover, electroporation, sonoporation, iontophoresis, and microneedles are physical methods that may be applied so as to deliver products into the cells (Ain et al., 2021).

Gene administration may be *ex vivo*, involving cells harvested from the patient. Gene administration can also occur *in vivo* while delivering the vector carrying the correct gene to the patient via transfusion. In the last case scenario, the vector must be designed with a specific tropism for the target organ. Another means of administration consist of *in situ* injection into tissues or topical application, such as on the skin (Papanikolaou and Bosio, 2021).

#### Gene Insertion (Augmentation)

**FIGURE 4:**
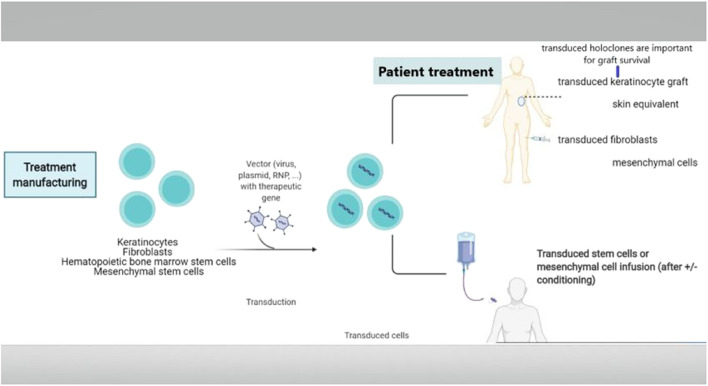
Gene replacement therapy. Delivering the correct gene to the patient is called gene augmentation. A vector is used to transduce a recombinant gene into the patient’s cells, including keratinocytes, fibroblasts, hematopoietic stem cells, mesenchymal stem cells, or iPSC. Usually, this is carried out *in vitro*, which is then followed by transplantation of the transduced cells. Moreover, several vectors even allow for *in vivo* transduction.

Gene transfer refers to the process of inserting the correct gene into the cells, in addition to the defective gene, thereby augmenting the alleles for the respective gene. This technique was first used for PID’s (Figure 4). If stem cells are transfected, there is the possibility of life long gene insertion, especially after transfecting with viruses that integrate into the genome.

The first genodermatosis studies were carried out *ex vivo* in *TGM1*-deficient ARCI keratinocytes (Choate et al., 1996). Later on, an *ex vivo* investigation was conducted using corrected *LAMB3*-deficient keratinocytes that originated from a patient suffering from JEB. This was followed by skin grafting over a small area (Mavilio et al., 2006), resulting in long-term graft survival (De Rosa et al., 2014). However, the real breakthrough came up with a JEB patient for whom 80% of the body surface were treated following *ex vivo* gene transfer to keratinocytes (Hirsch et al., 2017). Cultured autologous keratinocytes were transfected with retroviruses expressing full-length *LAMB3.* These keratinocytes were subsequently expanded in order to obtain large sheets of keratinocytes. They were then used to graft about 80% of the patient’s body surface (Hirsch et al., 2017). After 21 months, the skin displayed a normal appearance. It was demonstrated that holoclones (colony-forming stem cells with a higher growth potential than a mero- and paraclone, not containing differentiated cells) turned out to be the progenitor cells that were responsible for sustained skin regeneration, as the integration patterns of mero- and paraclones over time resemble more and more those of holoclones.

Important concerns for gene insertion, however, are vector-induced off target effects and malignancy risk, in addition to viral packaging capacity and immune reactions, especially in patients without any protein expression. Therefore, highly branched poly*β*‐amino esters (HPAE) appear to be promising alternatives, designed to deliver full length *COL7A1* in the form of polyplexes like AP103, which has been granted orphan drug status for DEB treatment.

Similar projects have already been initiated for other conditions, including NS (Di et al., 2011) (Di et al., 2019) (for a review see (Ain et al., 2021) (Jayarajan et al., 2021)), porphyrias (Bustad et al., 2021), and DEB (Eichstadt et al., 2019). For the latter, experiments in engineered epidermal-dermal skin substitutes suggest that expression of the *COL7A1 gene* in both keratinocytes and fibroblasts is most likely necessary so as to produce structurally normal anchoring fibrils. (Supp et al., 2019). This is presently further evaluated in clinical trials.

#### Gene Editing

**FIGURE 5:**
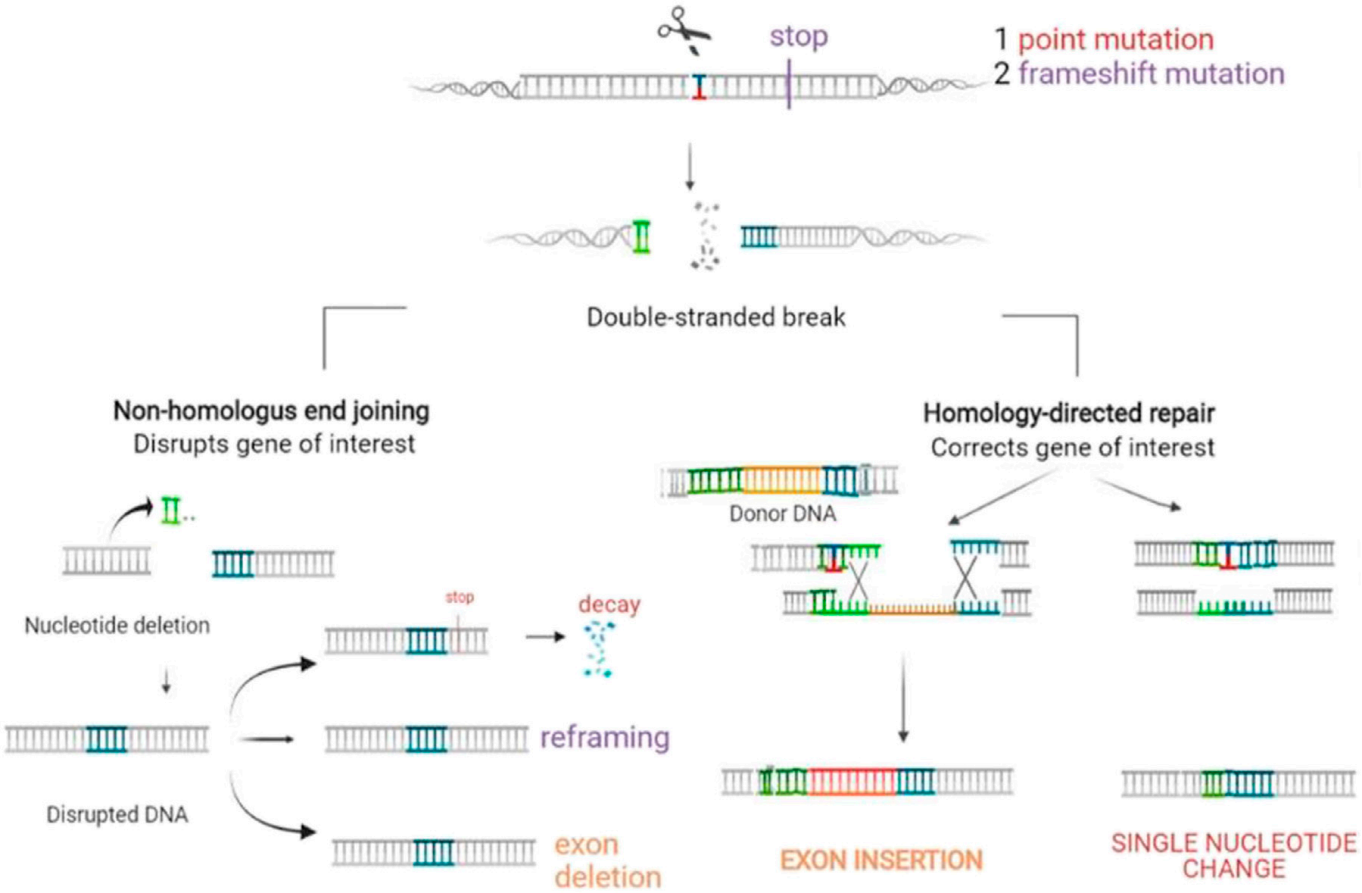
Gene editing. Gene editing offers diverse possibilities adapted to the genetic defect to be corrected. A vector is then required to enter the tools into the cells. The first step is to carry out a double strand break using nucleases. These breaks will be repaired by the error prone non-homologous end joining (NHEJ) with loss of a small number of nucleotides, which may result in non-expression of the aberrant protein. This option represents a therapeutic opportunity for diseases where haploinsufficiency carries an advantage in the case of over-expression of structurally aberrant proteins, which occurs in most keratinopathies. It may also have the potential of reframing in the case of indels or of deleting a codon or exon with the mutation. Repair by the homology directed repair (HDR) pathway enables incorporating the desired sequence alterations based on a donor DNA template with a normal sequence.

Genome editing is a recent type of genetic engineering, which is still largely in the preclinical phase. With this new technology, DNA is inserted, deleted, modified, or replaced in the genome of a living organism (Figure 5), thereby enabling more precise interventions. Except for base editors that are treated later in the paper, gene editing consists of carrying out double-strand breaks using nucleases including zinc finger nucleases (ZFNs), mega-nucleases, transcription-activator like effector nucleases (TALEN), as well as the most recently introduced clustered regularly interspaced short palindromic repeats (CRISPR/Cas9 and his mutant CRISPR/Cas9 variant). These double-strand brakes (DSB) will likely be repaired by taking advantage of the cell’s intrinsic repair pathways, using either the fast, yet error prone non-homologous end joining (NHEJ), or the homology directed repair (HDR). While the latter appears to be less efficient, it is certainly more precise, enabling the precise incorporation of desired sequence alterations. These nucleases must be designed in a way that enables them to precisely target the DNA sequence (or RNA) designed to be altered, which is carried out with tandem arrays of 33–35 amino acid repeats that either bind a specific nucleotide in TALEN or a guide RNA complementary to the targeted DNA in CRISPR [for a review see (March et al., 2018)]. A vector is required to enable the tools to enter into the cells. The choice of therapeutic strategy largely depends on the mutation type to be corrected [for a review see (March et al., 2020)].

##### Disruption

of pathogenic alleles can be obtained by generating a single DSB, which is then followed by error-prone NHEJ-based repair, thereby inducing indels into the target gene. This frequently provokes frameshifts within the target allele, likely to induce premature termination codons (PTCs) followed by nonsense mediated *m*-RNA decay (NMD), without any pathogenic protein formation.

This technique is specially suitable for managing dominant-negative genetic variants causing skin diseases (March et al., 2020), which is the case for most keratinopathies, in which the phenotype expression results from the abnormal protein produced *via* a modified allele, yet not from a protein reduction, should the allele be haplo-insufficient.

Two proof-of-concept studies based on a similar, non-allele-specific protocol, were developed. The first was focused in EB simplex with mutant *KRT5,* targeting exon 1 of wild type and mutant *KRT5* alleles (Aushev et al., 2017); the second concerned epidermolytic ichthyosis (EI), using a TALEN nuclease, designed to target a region of *KRT10*, upstream of a PTC known to induce a genetic knockout (March et al., 2019). Nevertheless, this technique required that, after editing, a selection of those keratinocyte clones where only the mutated allele was disrupted was performed, these may then be further expanded in culture. Both studies obtained high gene disruption percentages both in immortalized (Aushev et al., 2017; March et al., 2019) and in mutant keratinocytes from patients (March et al., 2019). Due to the selection required, this technique is only suitable for *ex vivo* procedures.

In addition, two mutation-specific targeting studies were conducted. Of these, one was an *in vivo* study conducted in *KRT9* mutant transgenic mice using CRISPR-Cas9, which turned out to be successful (Luan et al., 2018). Indeed, *KRT9* mutations are involved in epidermolytic PPK. A second study was conducted *ex vivo* in dominant DEB. Induced pluripotent stem cells (iPSCs) were successfully transduced, with loss of expression of the aberrant C7 obtained (Shinkuma et al., 2016). To be effective, a careful design is needed, which should seek complete destruction of the affected allele, thereby resulting in a complete absence of the aberrant protein.

##### Exon Reframing

Restoration of the reading frame may constitute a solution for frameshift inducing variants, provided that the amino-acid divergence induced by the correction results in a semi-functional protein. In this case, the same technique as for disruption may be applied, given that approximately one-third of all nuclease-induced indels lead to reading frame restoration. This approach appears useful for recessive mutations where expression of slightly modified protein variants replacing null variants is likely to provide some therapeutic alleviation. Preclinical studies have been conducted in the setting of recessive DEB. Their results demonstrated that the c.6527insC mutation of *COL7A1*, which is rather common in Spanish patients, appears suited for exon reframing (Chamorro et al., 2016; Mencía et al., 2018) as well as the c.5819delC, (Takashima et al., 2019).

##### Exon Deletion

Dual CRISPR/Cas9 targeting (with CRISPR/Cas9 sgRNA) can be used for highly efficient excision of the intervening sequence. Nevertheless, this technology carries a high risk for off target damage. Hence, an extremely careful design is required, thereby enabling this technology to exclusively target the desired DNA sequence comprising the pathogenic variant. Nevertheless, this technology appears particularly promising, especially if hotspot mutations are present in a particular exon. Indeed, this provokes protein decay and, if skipping of the exon gives rise to a functional shortened protein, this results in a less severe clinical phenotype. Preclinical studies deleting exon 80 have been carried out *in vivo* in *COL7A1* c.6485G > A mut/mut mice (Wu et al., 2017) and in keratinocytes of Spanish patients with the recurrent c.6527insC as well (Bonafont et al., 2019)**.** Exon skipping with antisense oligonucleotides (see next session) however is already in a more advanced developmental stage, whereas this technology is not associated with the possibility to result in permanent recovery.

##### Exon/Gene Insertion

Exon/gene insertion approaches utilize HDR machinery, requiring a repair template that harbours left and right homology arms, which bind with the DNA fragment surrounding the insertion place, so as to enable precise insertion of large correct DNA fragments. In comparison with gene augmentation, in which incorporation of receptor DNA occurs at random, these techniques carry the advantage of abrogating the risk of insertional mutagenesis and transgene expression. Because HDR is only active during late S/G2 replication phase, this technique appears to be poorly efficient, thus requiring selection and cultivation of correctly repaired cells afterwards. However, in theory, there is no restriction on either disease or mutation.

Preclinical studies have been performed for DEB (Sebastiano et al., 2014; Osborn et al., 2018), but in JEB as well (Benati et al., 2018).

##### Homologous Recombination

Directly reverting a disease-causing variant in a gene via a single-nucleotide change may be reached using homologous recombination. This is a personalized, mutation specific repair, based on a repair template during HDR, but without integrating the whole exon. The template can serve for different mutations that cover the complementary template.

This technique has been evaluated in XP fibroblast cell lines carrying a homozygous deletion in exon 9 of the XPC gene (Dupuy et al., 2013). Although a correction in only 2.5% of the cells was attained, the investigators suggested this may be sufficient for clinical efficacy. Other preclinical studies demonstrating the feasibility of this technique were conducted in recessive DEB (Izmiryan et al., 2016; Izmiryan et al., 2018; Kocher et al., 2019), along with another study demonstrating its feasibility in EBS (Kocher et al., 2017).

##### Base Editing

Base editing is a new technology that enables the direct, irreversible conversion of a specific DNA base into another at a targeted genomic locus. Current base editors contain a catalytically impaired CRISPR–Cas nuclease (that cannot make DSBs), serving to guide the binding, which is fused to a single-stranded DNA deaminase enzyme and, in some cases, to proteins that manipulate the DNA repair machinery (Anzalone et al., 2020). Hence, this technique does not rely on double-stranded DNA breaks. Instead, this approach uses enzymes designed to precisely rearrange some of the atoms in one of the four bases that structure either DNA or RNA. Two main classes of base editors have been developed to date including cytosine base editors (CBEs), which catalyze the conversion of C•G base pairs into T•A base pairs, in addition to adenine base editors (ABEs), which catalyze A•T–G•C conversions. Upon Cas binding, hybridization of the guide RNA spacer to the target DNA strand causes displacement of the PAM-containing genomic DNA strand to form a ssDNA R-loop (Anzalone et al., 2020), while within this loop, bases can be edited. A first proof of concept study was conducted in RDEB, providing promising results (Osborn et al., 2020). The technique’s advantage is that it is template independent, giving rise to fewer indels, while enabling high correction efficacy. However, there is a risk for off target damage. As a result, this technique is only suitable for point mutations, provided they can be targeted using CRISPR-Casp. Base editing is suitable not only for correcting single base mutations but also for gene disruption (March et al., 2020).

#### Natural Gene Therapy

**FIGURE 6:**
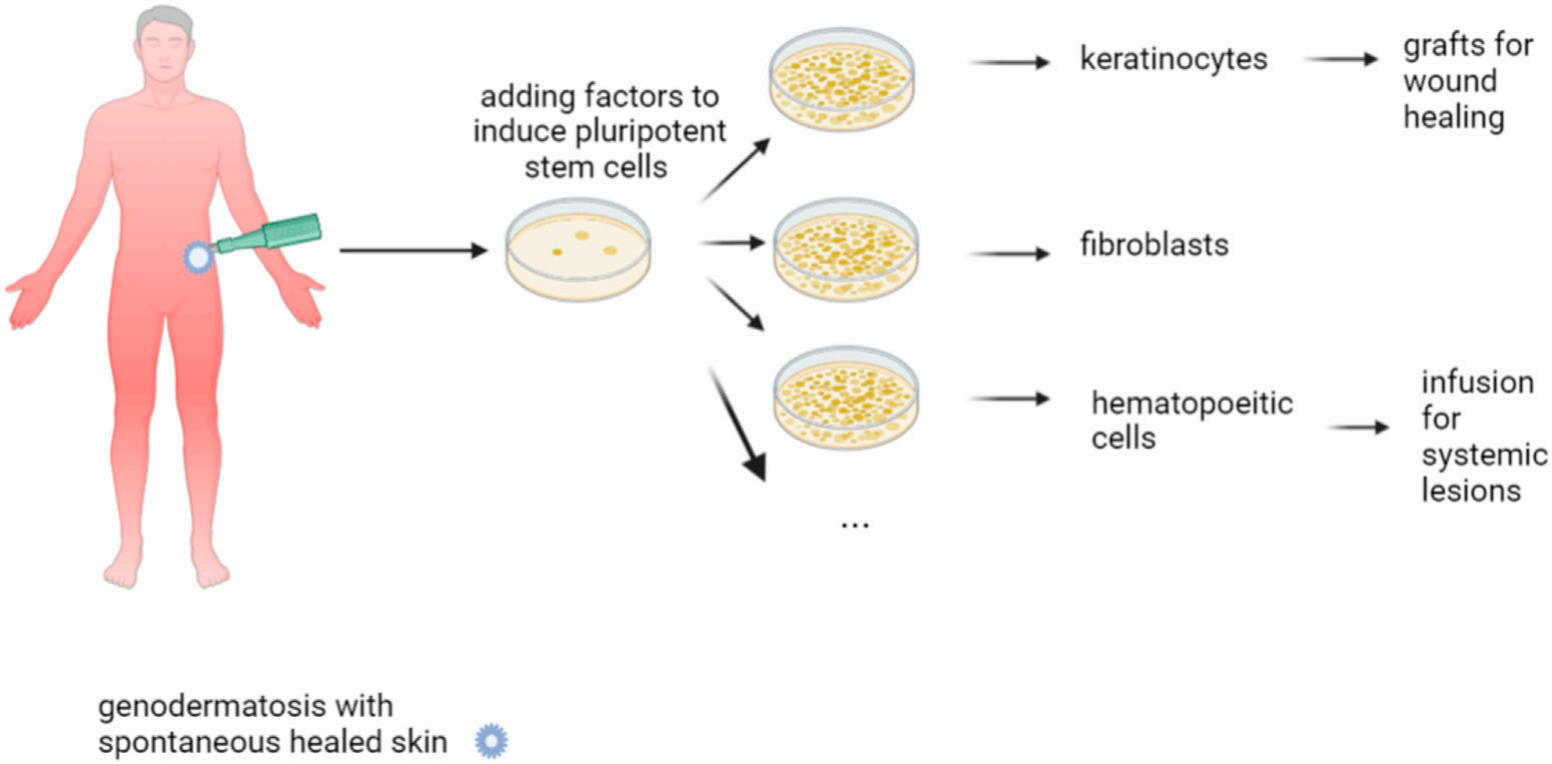
Natural gene therapy. Natural gene therapy is based on the appearance of normal skin within genetic aberrant one. This is caused by newly occurring mutations within genetically aberrant skin, which correct the defect, at least to some extent. This phenomenon of revertant mosaicism can be exploited for therapy by grafting this revertant skin into wounds. Reprogramming the revertant keratinocytes into iPSC and proliferation in culture do offer an inexhaustible source of cells, which can then be converted into keratinocytes or hematopoietic cells that are either transplanted or transfused into the patient.

Nature sometimes corrects a disease phenotype, and this phenomenon is called revertant mosaicism. It is not rare and well known in plant biology. In humans, this phenomenon appears to be common in self-regenerating organs like skin, blood, and liver, being particularly frequent in certain diseases. Concerning the skin, this condition was first described in JEB, presenting as patches of normal texture and tan, which never blister (Jonkman et al., 1997), with at least 35% of JEB carrying revertant patches (Jonkman and Pasmooij, 2012). Subsequently, this entity has been detected in dystrophic and simplex EB, Kindler syndrome, ichthyosis with confetti (IWC), keratitis-ichthyosis-deafness syndrome, dyskeratosis congenita, Bloom syndrome [reviewed in Jonkman and Pasmooij (2012), Pasmooij et al. (2012a)], and loricrin keratoderma, as well (Suzuki et al., 2019). In addition, the condition is commonly found in primary immunodeficiency disorders like Wiskott-Aldrich syndrome (Davis et al., 2010). The mechanisms underlying revertant mosaicism appear to be rather diverse, including gene conversion, intragenic crossover, mitotic recombination, back mutation, and second-site mutations (such as indels) (Pasmooij et al., 2012a). Ichthyosis with confetti (IWC) is caused by frameshift mutations, affecting the tail domain of the affected protein, either K10 or K1. The characteristic confetti lesions are caused by loss of heterozygosity caused by mitotic recombination (Guerra et al., 2015). Hence, there are mutations that promote reversion. In DEB and JEB patients with different small normal patches, genetic analysis revealed distinct gene conversion events (Pasmooij et al., 2005; Pasmooij et al., 2012b). Given this scenario, corrected cells would exhibit a proliferation advantage, leading locally to complete or partial phenotype reversion (Lai-Cheong et al., 2011). The triggers inducing these second hits are not yet well understood, with Sun exposure suggested for IWC. This spontaneous occurring phenomenon offers excellent therapeutic opportunities if these revertant cells expand *in vivo*, which is referred to as natural gene therapy. The technique’s advantages are its lack of immunogenicity and rejection risk; in addition, it does not require expensive genetic engineering techniques that are associated with a genotoxicity risk. Punch grafts (Gostyński et al., 2014) or cultured epidermal autografts (Matsumura et al., 2019) from a donor site with reverted skin onto wounds would, indeed, be the simplest method. Their survival on long-term likely depends on the presence of a sufficient number of reverted cells within the culture and, as learned from the skin graft trial using transgenic autologous stem cells in JEB (Hirsch et al., 2017), as well, on the presence of a sufficient number of holoclone stem cells, which were not seen in most biopsies taken. Reprogramming different cell types into iPSCs (Tolar et al., 2014) offers further opportunities. Tolar et al. (Tolar et al., 2014) succeeded in reprogramming revertant keratinocytes into iPSC and letting them proliferate. Subsequently, they succeeded in deriving these iPSC into hematopoietic cells, as well as epidermis-like keratinocyte layers, with C7 deposition. This outcome does open novel possibilities as these revertant hematopoietic cells could be employed for autologous HCT, without any need for pre-transplant conditioning, and with less toxicity. Moreover, as the iPSC are an inexhaustible source of keratinocytes, this technology offers tremendous possibilities in the context of local wound healing in patients carrying spontaneous revertant patches (Figure 6).

#### RNA-Based Techniques

Different techniques are being developed using exogenously-administered RNA-molecules or manipulating endogenous mRNA through editing or readthrough [for a review see (Bornert et al., 2017)]. It is an alternative technique for genome editing aimed at the same type of restoration on protein level and applicable *in vivo*. Given that the changes are performed at the RNA level, they are, however, not long-lasting.

Small interfering RNAs (si-RNA) are small double-stranded RNAs, which target mRNA for degradation and abolishing protein production (knock-down), by using the endogenous RNA silencing complex (RISC) (Uitto et al., 2012; Bornert et al., 2017). It constitutes an alternative to gene disruption using editing techniques. To date, most research has been conducted in the field of keratinopathies. In pachyonychia congenita gene disruption was proven feasible using a mutation specific design, while destroying the mutated allele (Leachman et al., 2010). The injected lesions displayed less hyperkeratosis, however the pain the injections caused definitely restricted its use. To overcome limitations of mutation specificity, another approach consisting of eliminating both mutated and wild type mRNA has also been considered. This approach can be used if keratins are interchangeable, such as the three paralogs *KRT6A*, *KRT6B,* and *KRT6C* encoding the three K6 isomorphs, including K6a, K6b, and K6c, all of which are expressed in palmoplantar skin (Leachman et al., 2010). Recently, a study has been completed, involving three patients with a mutation in *KRT6A* targeted by the si-RNA TD101 (NCT00716014). This knock-down strategy is also under investigation for dominant dystrophic EB (Pendaries et al., 2012). A si-RNA, targeting the enzyme aminolevulinic acid synthase 1 (Givosiran), which catalyses the formation of delta-aminolevulinic acid, is also currently available. This is meant to prevent repetitive acute abdominal pain attacks occurring in certain porphyria subtypes, including some cutaneous forms like variegate porphyria, where acute abdominal pain is a presenting sign. The common mechanism is the accumulation of haem synthesis intermediates, mainly comprising delta-aminolaevulinic acid (Bustad et al., 2021).

**FIGURE 7:**
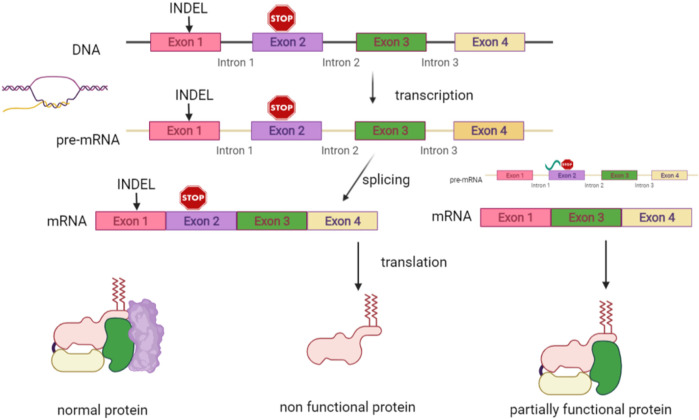
RNA-based therapies. Interference is additionally possible at RNA level. Antisense oligonucleotides (AONs) are designed to hybridize during pre-RNA splicing to the region of the exon with a mutation. This results in skipping of this exon and production of an internally deleted protein, which is designed to be still functional. It is applicable to diseases where the presence of a shorter protein provokes a less severe phenotype than in the case of its totally or partially absence.

Antisense oligonucleotides (AONs) are presently considered an attractive class of compounds. This is a personalized treatment, given that the oligonucleotides are designed to target specific disease-causing mutations, if localized on in frame exons. AONs hybridize during pre-RNA splicing, while hiding it, which results in skipping of the exon with the mutation (Bornert et al., 2017). An internally deleted protein is thereby produced, which is aimed to be still functional, hence applicable for diseases where the presence of a shorter protein provokes a less severe phenotype, as compared to its (partially) absence (Figure 7). Exon skipping is a technique that reached clinical application in Duchennes muscular dystrophy. Pre-clinial studies have so far been conducted in DEB, given that the *COL7A1* gene is considered to be a good candidate, owing to its large size, counting 117 exons. Indeed, most of its exons are rather short and primarily in frame, enabling them to be removed without disturbing the open reading frame and with conservation of most of the protein. Moreover, a study focusing on clinical and molecular DEB patient data revealed that recessive variants in exons that are affected by exon skipping, as part of natural low level alternative splicing events, generally represent relatively mild phenotypes within the clinical RDEB spectrum, whereas for dominant pathogenic variants in exons undergoing natural exon skipping, it seems to make less difference (Bremer et al., 2019). In DEB models, it has been proven for exons 13, 70, 73, 80, and 105 that skipping of one exon allows for the formation of a slightly shorter, still functional protein that should improve the phenotype (Goto et al., 2006; Bornert et al., 2016; Bremer et al., 2016; Bornert et al., 2021). A first study using an AON targeting exon 73 in a carbomer-based hydrogel (Q313) in both dominant and recessive DEB is actually recruiting patients (NCT03605069). Systemic administration, as practiced in Duchennes muscular dystrophy, would offer the possibility to additionally treat extra-cutaneous symptoms, provided such application is tolerated on the long-term (Bremer et al., 2016). Exon skipping is also under investigation in the settings of *COL17 A1* mutations causing junctional EB (Condrat et al., 2018). Proof of its feasibility is the spontaneous occurrence of areas of normal skin in a JEB patient being a carrier of compound heterozygous (a frameshift and a nonsense) pathogenic variants in *COL17 A1.* This “normal” skin showed an additional splice mutation, resulting in skipping of the exon and formation of a truncated, yet functioning, protein (Kowalewski et al., 2016). As AONs are rather short-lived, repeat treatments are likely necessary, whereas there are still some concerns with respect to long-term safety (Hilhorst et al., 2019; Nguyen et al., 2019).

Mutations creating a new splice site may likewise be a good target for AONs, especially if located deeply intronic, enabling specific splicing without off target damage. These mutations prevent the splicing machinery to recognize the cryptic splice site and, as demonstrated in a preclinical study in NF1, enable restoring normal splicing (Pros et al., 2009). An AON targeting the hypomorphic *FECH* polymorphism IVS3-48C > T, which redirects the cryptic to the physiologic splice site, is currently under investigation for EPP (Erwin and Balwani, 2021).

Spliceosome-mediated RNA *trans*-splicing (SMaRT) is a technique replacing the pre-mRNA fragment, containing the disease-causing variant, thereby resulting in a hybrid full-length wild-type mRNA (Bornert et al., 2017). Using the cell’s splicing machinery, an exogenous piece of mRNA replaces the fragment containing the pathogenic variant, starting from the splice site preceding the mutation. Preclinical studies have been conducted in an *in vitro* disease model, using fibroblasts from EB simplex with muscular dystrophy patients, carrying variants in the *plectin* gene. In this model, wild type allele expression could be increased by 58.7% (Wally et al., 2008). Proof of concept was similarly obtained in a DEB model (Fritsch et al., 2009; Murauer et al., 2011; Morgan et al., 2013).

Micro RNAs (miRNAs), which remain untranslated, are endogenous post-transcriptional modulators of gene expression with critical functions in health and disease. Most probably, together with DNA/RNA methylation, they represent an important epigenetic factor regulating gene function (López-Jiménez and Andrés-León, 2021) (Wagner et al., 2021). There is some evidence that they might explain at least to some extent, several phenotypic variations of genodermatoses within individuals harboring the same genetic background. A recent study revealed miR-125b to be upregulated in HHD patients suffering from clinical symptoms (Manca et al., 2011). The authors suggest that oxidative stress-mediated induction of miR-125b (Manca et al., 2011) plays a specific role in the HHD pathogenesis, specifically by regulating the expression of proteins involved in Notch signaling. Indeed, this pathway plays a key role in keratinocyte proliferation and differentiation and possibly in clinical HHD manifestations, as well. In EB, miRNAs play a major role in fibrosis (miR-29b and miR-145) and SCC development (miR-10b) (Wagner et al., 2021). In recessive DEB, the miR-29 levels are decreased, resulting in increased miR-29 target mRNAs, including pro-fibrotic extracellular matrix collagens. It is likely to be an essential regulatory mechanism for C7 production and TGF-β-mediated fibrosis inhibition (Vanden Oever et al., 2016). MiRNAs are a recent research topic, which could bring upon novel therapeutic options in the future.

Other research topics concerning RNA-based techniques are transcript transfer, which introduces *in vitro* transcribed mRNA into the cell in order to translate it into wild type proteins, as well as the use of mRNA transcripts encoding nucleases for genome editing (Bornert et al., 2017; Bustad et al., 2021).

## Prevention Strategies

It is crucial that each family with an affected member receives appropriate genetic counselling regarding the variability of the disease phenotype, its genetic transmission, and the possibilities of prenatal or preimplantation genetic testing. Special educational programmes are currently available for several genodermatosis groups. The support provided by a psychologist and by patient organisations are extremely valuable and are designed to accompany the families that are confronted with such rare diseases.

Despite all these upcoming treatment possibilities, prevention of recurrence in future pregnancies remains crucial, based on techniques of prenatal or preimplantation genetic testing, with the implantation of a non-affected embryo as an option. However, this domain is beyond the scope of this article, but we refer to comprehensive reviews on these topics (Vermeesch et al., 2016; Jónsson et al., 2018; Antonarakis, 2019; Che et al., 2020).

## Conclusion

Genodermatoses have long been considered as diseases for which effective treatments were almost non-existent, with very little hope for inventive progression. However, given that the underlying mutations are progressively unravelled and along with translational research generated, more possibilities are currently at the horizon.

These mutations may cause dysfunction of biochemical pathways thereby offering opportunities to interact with dysfunctional paths. One approach consists of repurposing drugs, which are already well-known and have been investigated for other pathologies. If inflammatory pathways are triggered, targeted biological therapies can be applied.

However, modern technologies are additionally offering new inventive tools, enabling us to intervene with the real cause of genodermatoses and thus either restore or replace the affected gene or gene-product. Some of these new techniques likely enable very precise corrections. Besides, if DNA in stem cells can be corrected, then the dream of definitive correction comes close to reality. The first, *in vivo* trials, using genetically-engineered cells and tissues are currently under way. These sophisticated technologies are only possible in the event that a molecular genetic diagnosis is available. Moreover, these techniques require dedicated and specialised competences, subsequent care and follow-up. For some genodermatoses, such competences are only available in certain centres. Therefore, collaboration across Europe appears crucial, allowing us to offer our patients the best possible care, with the support of the European Union. More information is to be found here: https://ec.europa.eu/health/cross_border_care/overview_en.

In this article, we have shed light over different new avenues that are currently being investigated. Each of the above-mentioned genodermatoses poses its own challenges, in regard to its way of transmission, mutation type and localisation, and altered protein function. With respect to genome editing and RNA interference, these approaches constitute personalised therapies for which feasibility, lack of undesirable effects, and off target damage, in addition to cost/effectiveness, are likely to play a role in determining which of interventions is likely to be made available for clinical use.

Therefore, information on these conditions and prevention of these, at times, severe and life threatening diseases that dramatically impair the quality of life of the patients and their families, are of interest. An increasing number of centres offer the possibility of patient follow-up at specialised consultations for genodermatoses. This is instrumental in enabling a better follow-up, superior information, earlier diagnosis of potential complications, as well as the possibility to be directed to new treatment options that are at the horizon. While the future is not yet bright, there is at least some sunshine.

## Glossary

ABEs: adenine base editors
AEC: ankyloblepharon ectodermal defects cleft lip palate syndrome
AED: anhidrotic ectodermal dysplasia/HED hypohidrotic ectodermal dysplasia
ARCI: autosomal recessive congenital ichthyosis
BCC: basal cell carcinoma
BM: bone marrow
C7: type VII collagen
CARD 14
CBEs: cytosine base editors
CRISPR/Cas9: clustered regularly interspaced short palindromic repeats
CS: Cockayne Syndrome
CXCR4: C-X-C Motif Chemokine Receptor 4
DEJ: dermo-epidermal junction
DSB: double strand bond
EB: epidermolysis bullosa (JEB junctional EB; DEB dystrophic EB)
EBS: epidermolysis bullosa simplex
EDA: ectodysplasin A
EGFR: epidermal growth factor receptor
GLA
GOF: gain of function
HB-EGF: heparin-binding epidermal growth factor-like growth factor
HDR: homology directed repair
HHD: Hailey-Hailey disease
HMGB1: high mobility group box 1 protein
HSV: human herpes virus
LOF: loss of function: loss of function
HI: Harlequin ichthyosis
HPAE: highly branched poly*β*‐amino esters
ID: intradermal
Indels: insertions or deletions
IV: intravenous
JAK
LAMB3
LNPs: lipid based nanoparticles
LOF: loss of function: loss of function
MAPK: mitogen-activated protein kinase
MSC: mesenchymal stem cells
NHEJ: non-homologous end joining
NF1: neurofibromatosis type 1
NF-κB: nuclear factor-κB
NIPAL4
NMD: non sense meditated *m*-RNA decay
NOS2: nitric oxide synthase
NS: Netherton syndrome
OCA: oculocutaneous albinism
PDGFRα: platelet-derived growth factor receptor-α
PPK: palmoplantar keratoderma
PRP: pityriasis rubra pilaris
PTC: premature termination codons
SDF1a: stromal cell derived factor 1a
SERPINB7
SMO: smoothened oncogene
SCC: squamous cell carcinoma
STAT
TALEN: transcription-activator like effector nucleases
TGF-α (−β): transforming growth factor α (b)
TGM-1: Transglutaminase 1
tNG: thermoresponsive nanogels
tRNA: transfer RNA
TRPV3: *Transient Receptor Potential Vanniloid-3*
XP: xeroderma pigmentosum (XPC type C XP)
ZFNs: zinc finger nucleases
